# Are there any limits to perform segmentectomy for non-peripheral early-stage non-small-cell lung cancer?

**DOI:** 10.1093/icvts/ivaf059

**Published:** 2025-03-14

**Authors:** Apostolos C Agrafiotis, Paul E Van Schil

**Affiliations:** Department of Thoracic and Vascular Surgery, Antwerp University Hospital, Edegem (Antwerp), Belgium; Department of Thoracic and Vascular Surgery, Wallonie Picarde Hospital Center (Centre Hospitalier de Wallonie picarde—CHwapi), Tournai, Belgium; Department of Thoracic and Vascular Surgery, Antwerp University Hospital, Edegem (Antwerp), Belgium; Antwerp Surgical Training And Research Center (ASTARC), University of Antwerp, Wilrijk (Antwerp), Belgium

**Keywords:** segmentectomy, sublobar resection, surgical margin

We read the article by Yang *et al.* with great interest. In this retrospective observational study, the authors enrolled 850 patients with clinical stage IA1-IA2 non-small-cell lung cancer (NSCLC) who underwent segmentectomy [1]. In an era when sufficiently powered randomized controlled trials have challenged the superiority of lobectomy over sublobar resection, new insights into segmentectomies have emerged [2–4]. The authors utilize the tumour distance index to classify the patients into peripheral (*n* = 576) and non-peripheral (*n* = 274) groups. Disease-free survival (DFS) and overall survival (OS) were compared between the two groups. Although there was no statistically significant difference between the groups in 5-year DFS and 5-year OS, a higher margin recurrence was observed in the non-peripheral group. Additionally, patients with a non-peripheral solid lesion had worse 5-year DFS and an increased margin recurrence rate. In general, the margin distance was ≥2 cm or greater than the maximum tumour diameter, and a mandatory frozen section confirmed negative margins. However, the non-peripheral group had a smaller margin distance [24.3 mm (19.4–33.3) versus 29.7 mm (20.3–40.0); *P* < 0.001], confirming that achieving an adequate margin distance for non-peripheral segmentectomy is technically challenging. The importance of preoperative 3D planning in assessing the feasibility of obtaining sufficient surgical margins should be emphasized. We agree with the authors that if the planned resection margins appear unsafe, a lobectomy may be the better choice to obtain a complete resection with good long-term results.

Surgical margin distance was also specifically studied in a secondary analysis of the randomized CALGB 140503 study, showing no difference in local recurrence rate and OS in sublobar resection comprising wedge resection and segmentectomy, compared to lobectomy [4]. In the sublobar resection group, 13.7% local recurrences were observed. Surprisingly, neither surgical margin distance nor margin-to-tumour size ratio was predictive of local recurrence-free survival or OS, although these parameters were not available in the whole patient population [oral presentation at World Conference of Lung Cancer 2024, San Diego, CA, USA, abstract 2486, MA 03.10]. In contrast, high-grade adverse events negatively influenced DFS and recurrence-free survival, but not OS. Locoregional and distant recurrence-free survival were also affected but not significantly [oral presentation at World Conference of Lung Cancer 2024, San Diego, CA, USA, abstract 3081, MA 03.11]. In this way, prevention of high-grade adverse events is mandatory to reduce local recurrent disease and maximize survival.

In the same direction as the present ICVTS paper, a recently published study concluded that for carefully chosen individuals with central, small (≤2 cm) and radiologically solid-dominant NSCLC, segmentectomy may be a good treatment choice, offering local control and a prognosis similar to lobectomy [5].

In the present study, the median follow-up period was 6.7 years, and since margin recurrence seems to increase with time, longer follow-ups are warranted, especially as several local ablative treatment options are available for recurrent disease.

Other factors that can negatively influence survival rates, such as STAS (spread through air spaces), visceral pleural invasion and nodal upstaging, were not individually analysed in this study but may play a role in the occurrence of early recurrent disease.

In conclusion, segmentectomy should be carefully planned preoperatively to identify patients who could benefit from it. Extra caution should be applied in the case of non-peripheral and solid nodules. It is obvious that as experience with sublobar resection grows, as more complex segmentectomies are performed in more challenging localisations, new questions are raised, and further research is warranted to clearly delineate the limits of anatomical segmentectomy.

**Conflict of interest:** none declared.
